# Sensorineural hearing loss and mild cardiac phenotype caused by an *EYA4* mutation

**DOI:** 10.1038/s41439-018-0023-9

**Published:** 2018-08-22

**Authors:** Satoko Abe, Hidehiko Takeda, Shin-ya Nishio, Shin-ichi Usami

**Affiliations:** 1 Department of Otorhinolaryngology, Toranomon Hospital, 2-2-2 Toranomon, Minato-ku, Tokyo, 105-8470 Japan; 2 Department of Otorhinolaryngology, Shinshu University School of Medicine, 3-1-1 Asahi, Matsumoto, Nagano, 390-8621 Japan; 3 Department of Hearing Implant Science, Shinshu University School of Medicine, 3-1-1 Asahi, Matsumoto, Nagano, 390-8621 Japan

## Abstract

*EYA4* is a member of the vertebrate *eya* gene family of transcriptional activators and plays several roles in both embryonic and inner ear development. The majority of *EYA4* gene mutations are associated with autosomal dominant non-syndromic hearing loss (DFNA10). In addition, some mutations in this gene cause autosomal dominant syndromic hearing loss with dilated cardiomyopathy. *EYA4* is a rare cause of sensorineural hearing loss, and only a limited number of papers regarding mutations in this gene have been published. Thus, detailed clinical features remain unclear.

We conducted next-generation sequencing of a Japanese individual with progressive sensorineural hearing loss and identified an *EYA4* pathogenic variant. Pure-tone audiometry revealed bilateral, nearly symmetric, moderate sensorineural hearing loss in the low and middle frequencies. Minor abnormalities were observed on the patient’s electrocardiogram and echocardiography without any apparent symptoms. Next-generation sequencing is effective in elucidating the etiology of hearing loss, and the present findings suggested the possible phenotypic expansion of deafness caused by *EYA4* gene mutations.

Hearing loss is the most frequent sensory defect in humans, demonstrating different patterns of inheritance and involving a multitude of different genes. To date, ~180 non-syndromic hereditary sensory hearing loss loci and more than 100 corresponding genes have been identified^1^.

*EYA4* is a member of the vertebrate *eya* gene family of transcriptional activators that interact with other proteins, such as the sine oculis homeobox (SIX)  protein family, to ensure normal embryologic development^2^. Moreover, *EYA4* also plays several roles in the mature inner ear system^2, 3^. The majority of *EYA4* gene mutations are associated with autosomal dominant non-syndromic hearing loss (DFNA10) (OMIM:# 601316)^4–6^. In addition, some mutations in this gene cause autosomal dominant syndromic hearing loss with dilated cardiomyopathy (OMIM:# 605362)^7^. *EYA4* is a rare causative gene of sensorineural hearing loss and only a limited number of papers regarding mutations in this gene have been published. Thus, the complete picture with regard to the clinical features associated with these gene mutations remains unclear.

Remarkable recent advances in molecular genetics technology, particularly in the clinical applications of next-generation sequencing technology, have made it easier to identify disease-causing genes in a relatively short period and at low cost. Here, we report a Japanese individual with progressive sensorineural hearing loss (SNHL) in whom an *EYA4* pathogenic variant was identified.

A 43-year-old Japanese male from a non-inbred family presented to our hospital to clarify the cause of his hearing loss. His maternal grandmother, mother, maternal uncle, and sister also have late-onset hearing loss (after acquisition of language) (Fig. 1a). The proband’s family inheritance matches both autosomal dominant and maternal modes of inheritance. This study was approved by the local ethics committee, and written informed consent was obtained from each participating individual.

**Fig. 1:**
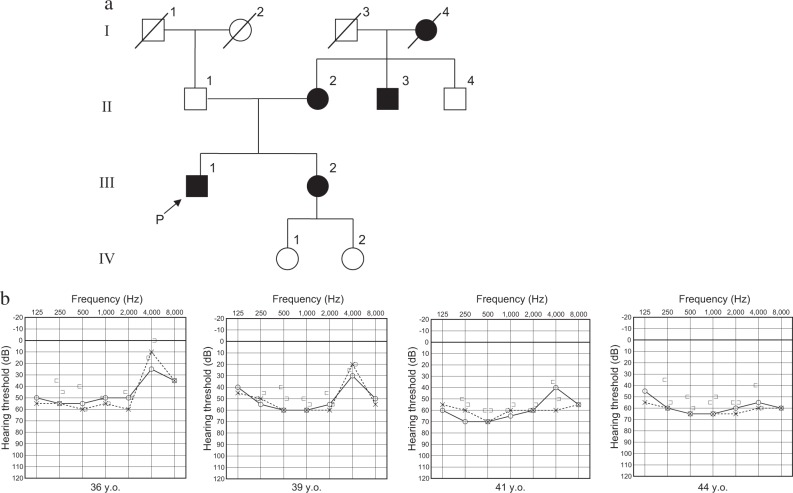
**a** Pedigree and **b** audiometric configurations of the proband (P). Closed symbols indicate affected individuals. The high frequency hearing thresholds of this patient progressed over the follow-up period and eventually showed flat-type moderate hearing loss

The patient’s hearing loss was first noticed during a checkup at age 26. He began wearing a hearing aid at age 36. Further deterioration in his hearing was recognized subjectively at 40 years of age due to loud noises associated with his work as a carpenter. At the age of 41, his tinnitus and hearing loss further worsened after exposure to loud firework explosions. No noticeable effects were observed upon treatment with various medications, including steroids. Clinically, he has no accompanying disease or disorder, but abnormalities in his electrocardiograms have been observed since his second decade of life.

Pure-tone audiometry revealed bilateral, nearly symmetric, moderate SNHL with hearing loss in the low and middle frequencies. The hearing loss was progressive, and an audiogram at age 43 showed hearing loss at all frequencies with a flat pattern (Fig. 1b). He has normal verbal function and experiences few problems with regard to linguistic communication when wearing hearing aids. He has no complaints of vestibular symptoms.

There was insufficient power in the kindreds to perform linkage studies, and we could not obtain consent from any of his family members. Thus, molecular diagnosis was performed using genomic DNA extracted from the peripheral blood of the proband alone. In the primary screening using the Invader assay^8^, no mutations were observed upon examination of 46 known mutations in 13 deafness genes. Secondary screening using comprehensive next-generation sequencing analysis for 63 genes reported to cause hearing impairment was performed as described previously^9^.

As a result of this genetic analysis, a heterozygous variation *EYA4*:NM_004100.4:c.1177 C > T:p.Q393X (in exon13) (hg19:chr6:g.133804239 C > T) was identified in the eyes absent 4 gene (*EYA4*, OMIM: *603550) in this patient. This result was confirmed by Sanger sequencing. This variation was previously reported as a pathogenic variant in a non-syndromic SNHL Korean pedigree^10^. This variant was absent in the 1,000 Genomes Project; 6,500 Exome Variant Server; 333 healthy controls in the Shinshu University Project; Human Genetic Variation Database and 2KJPN for the Japanese population databases; and the 1,000 controls of Iowa University. Only one case carrying this variant was present in the Exome Aggregation Consortium database (1/121,170 alleles, allele frequency = 0.000008). According to the pathogenicity classification based on the Association College of Medical Genetics (ACMG)^11^, we regard the p.Q393X variant as “pathogenic”.

Most patients harboring *EYA4* mutations present with non-syndromic SNHL, but some also experience dilated cardiomyopathy and heart failure accompanied by SNHL^7^. We, therefore, performed a detailed cardiac examination to confirm whether the patient had a non-syndromic phenotype. His electrocardiogram revealed an abnormal Q wave at V1 and V2 (Fig. 2a); however, the cardiologist evaluated it to be within the normal range. Echocardiography revealed mild mitral valve regurgitation from the left ventricle to the left atrium (Fig. 2b), but this was judged not to affect cardiac function. No problems were observed with the left ventricular dilatation or contractility. At present, the patient suffers subclinical heart disease not related to dilated cardiomyopathy.

**Fig. 2:**
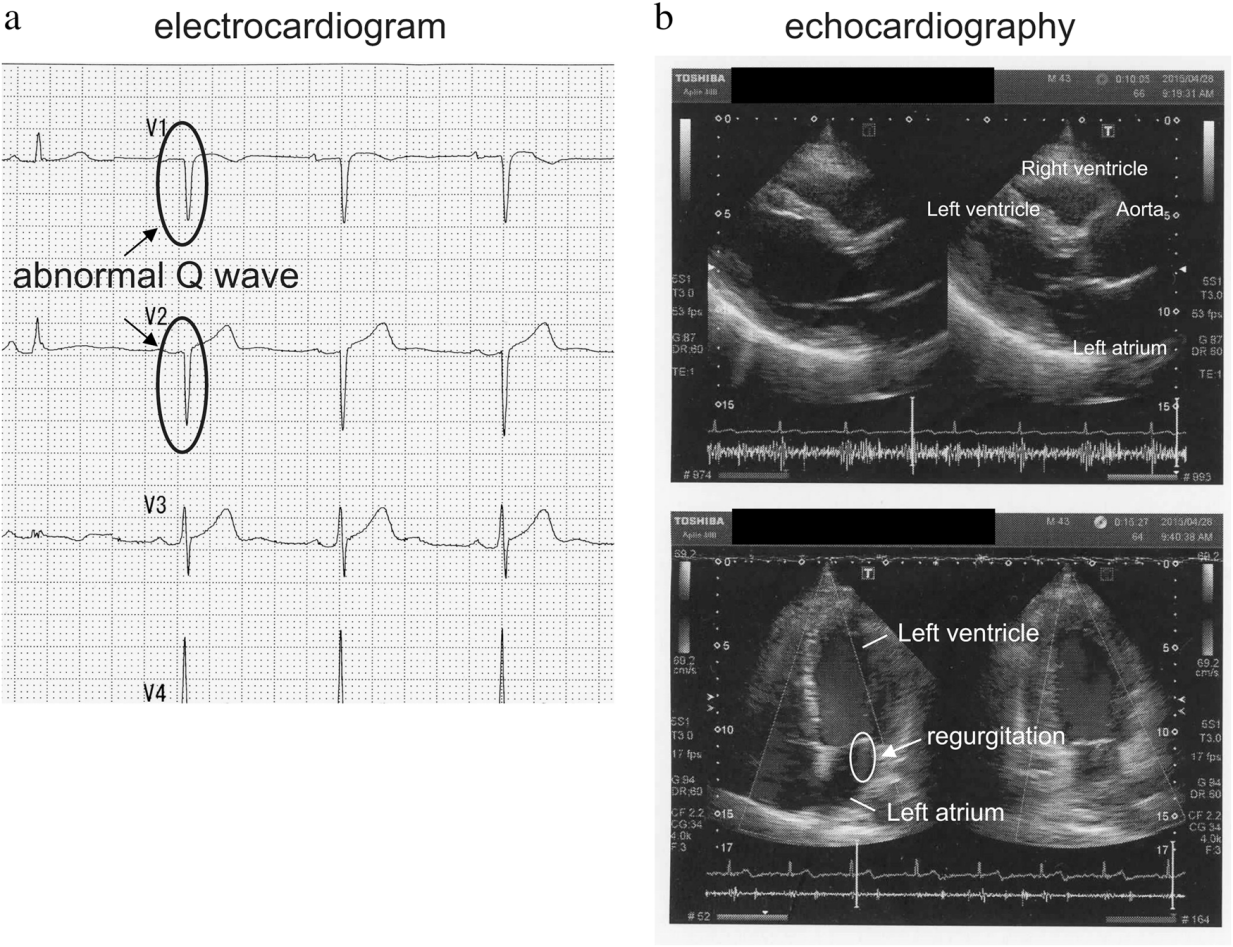
Results of the proband’s **a** electrocardiogram and **b** echocardiograph. The electrocardiogram revealed a minor abnormality in the Q wave at V1 and V2, while the echocardiography revealed mild mitral valve regurgitation from the left ventricle to the left atrium

Allele variants in *EYA4* cause inherited autosomal dominant hearing loss linked to the DFNA10 locus^4–6^ and are also associated with dilated cardiomyopathy in some cases^7^. Up to 13 mutations in *EYA4* have been reported in several autosomal dominant inherited hearing loss families with a similar phenotype worldwide^12–14^. Of the reported *EYA4* mutations, five are located in the eya-homologous region (HR), and eight are located in the variable region of the *EYA4* transcripts. The affected individual in this study carrying p.Q393X showed a similar progression of hearing deterioration to that observed in DFNA10 patients^15^. Further, the audiometric configuration of the previously reported Korean SNHL pedigree with the same mutation resembles that of the patient in this study^10^. It is therefore possible that both a mutational hot spot and founder effect are involved.

The dilated cardiomyopathy caused by *EYA4* gene mutations is a rare associated symptom. However, it should be noted that some cardiac abnormalities (such as aortic regurgitation, tricuspid regurgitation, inferior wall hypokinesis, or mild concentric hypertrophy) were detected by echocardiography in three of nine cases with *EYA4* mutations^6^. In terms of gene expression, *EYA4* is expressed in the heart and the inner ear^16^. Normal cardiac gene expression is sensitive to the concentration of EYA4-SIX complexes;^5^ therefore, a partial *EYA4* deficiency may induce small cardiac abnormalities despite a clinically normal status. Even in asymptomatic patients, it is possible to avoid overlooking potential cardiac degradation leading to more serious conditions, such as heart failure, by cardiac examination. The present data suggest that *EYA4*-associated hearing loss possibly exhibits an extensive spectrum of conditions ranging from minor abnormalities showing clinically normal heart function to dilated cardiomyopathy. It is hypothesized that the susceptibility to cardiomyopathy may be determined by the mutational position on the *EYA4* transcripts;^6^ however, no information of any genotype–phenotype correlation is currently available. Any such correlation will become clearer as data are accumulated in the future.

Next-generation sequencing is an effective strategy for elucidating the etiology of hearing loss with high genetic heterogeneity, and our findings will provide a basis for further exploration of the pathological mechanisms and appropriate counseling for patients with *EYA4* mutations.

In this paper, we report a Japanese patient with progressive sensorineural hearing loss carrying a rare autosomal dominant *EYA4* gene mutation. Minor abnormalities were observed on the patient’s electrocardiogram and echocardiography without any apparent symptoms. The present findings suggested the possible phenotypic expansion of deafness caused by *EYA4* gene mutations.

## Data Availability

The relevant data from this Data Report are hosted at the Human Genome Variation Database at 10.6084/m9.figshare.hgv.2366.

## References

[1] Hereditary Hearing Loss homepage. http://hereditaryhearingloss.org/ (2017).

[2] Wayne S (2001). Mutations in the transcriptional activator *EYA4* cause late-onset deafness at the DFNA10 locus. Hum. Mol. Genet..

[3] Wang L (2008). Eya4 regulation of Na+/K+-ATPase is required for sensory system development in zebrafish. Development.

[4] Pfister M (2002). A 4-bp insertion in the eya-homologous region (eyaHR) of *EYA4* causes hearing impairment in a Hungarian family linked to DFNA10. Mol. Med..

[5] Schönberger J (2005). Mutation in the transcriptional coactivator *EYA4* causes dilated cardiomyopathy and sensorineural hearing loss. Nat. Genet..

[6] Makishima T (2007). Nonsyndromic hearing loss DFNA10 and a novel mutation of *EYA4*: evidence for correlation of normal cardiac phenotype with truncating mutations of the Eya domain. Am. J. Med. Genet. A..

[7] Schönberger J (2000). Dilated cardiomyopathy and sensorineural hearing loss: a heritable syndrome that maps to 6q23-24. Circulation.

[8] Usami S (2008). The responsible genes in Japanese deafness patients and clinical application using Invader assay. Acta Otolaryngol..

[9] Miyagawa M, Nishio SY, Ikeda T, Fukushima K, Usami S (2013). Massively parallel DNA sequencing successfully identifies new causative mutations in deafness genes in patients with cochlear implantation and EAS. PLoS ONE.

[10] Kim YR (2015). Evaluation of the contribution of the *EYA4* and GRHL2 genes in Korean patients with autosomal dominant non-syndromic hearing loss. PLoS ONE.

[11] Richards S (2015). Standards and guidelines for the interpretation of sequence variants: a joint consensus recommendation of the American College of Medical Genetics and Genomics and the Association for Molecular Pathology. Genet. Med..

[12] Hildebrand MS (2007). A novel splice site mutation in *EYA4* causes DFNA10 hearing loss. Am. J. Med. Genet. A.

[13] Tan M (2014). Identification of I411K, a novel missense *EYA4* mutation causing autosomal dominant non-syndromic hearing loss. Int. J. Mol. Med..

[14] van Beelen E (2016). Audiometric characteristics of a Dutch DFNA10 family with mid-frequency hearing impairment. Ear Hear..

[15] De Leenheer EM, Huygen PL, Wayne S, Smith RJ, Cremers CW (2001). The DFNA10 phenotype. Ann. Otol. Rhinol. Laryngol..

[16] Nishio SY (2015). Gene expression profiles of the cochlea and vestibular endorgans: localization and function of genes causing deafness. Ann. Otol. Rhinol. Laryngol..

